# Academic performance and college students’ subjective well-being

**DOI:** 10.3389/fpubh.2026.1837740

**Published:** 2026-05-15

**Authors:** Qianqian Zhai, Qian Li, Shoaib Ahmed Wagan

**Affiliations:** 1 Faculty of Economics and Management, Langfang Normal University, Langfang, China; 2 College of Economics, Beijing Technology and Business University, Beijing, China; 3 Department of Rural Sociology, Sindh Agriculture University, Tando Jam, Sindh, Pakistan; 4 International Business School, Hainan University, Haikou, China

**Keywords:** academic performance, college student, gender, mental health, subjective well-being

## Abstract

Academic performance stands as a pivotal metric for schools in evaluating the proficiency of students, and it serves as a targeted objective for students to strive for. Based on the survey data of 327 undergraduate students, this paper empirically examines the association between college students’ academic performance and their subjective well-being. The results show that the average subjective well-being of college students is situated in the lower mid-range, with an observable tendency toward decline over time. The subjective well-being of college students is associated with both their absolute and relative grade point average (GPA). Specifically, the higher the GPA, the stronger students’ subjective well-being. However, students’ subjective well-being will be significantly reduced if their GPA is ranked lower. The association between academic performance and college students’ subjective well-being exhibits heterogeneity across gender, grade and place of origin. Specifically, the association between academic performance and subjective well-being is more pronounced among females and low-grade students than that of males and high-grade students, but dose not significantly differ by place of origin. We additionally find several other factors that affect students’ subjective well-being: joining school clubs, liking their majors, being extroverted and maintaining good health also significantly improves students’ subjective well-being. Given the cross-sectional design and single-institution sample, these findings should be interpreted as associational rather than causal.

## 1 Introduction

Within educational frameworks, fostering students’ subjective well-being is a paramount objective, complementing traditional goals centered on academic learning (1). Students’ subjective well-being has recently emerged as a critical issue due to its wide-reaching benefits for better performance in school and later as adults (2). However, our survey conducted in this research reveals that the overall level of subjective well-being among Chinese college students is suboptimal, situated in the lower middle tier, which is consistent with the survey results of German middle school students (3). In general, students in China outperformed in their subjects but suffered from a low level of well-being (4, 5). An expanding corpus of research indicates a downward trend in subjective well-being and a concurrent rise in mental health issues among the youth in recent decades (6), especially after the COVID-19 pandemic (7). The subjective well-being of students serves as the cornerstone for their learning and holistic development. Those with diminished subjective well-being are more susceptible to psychological maladies, including anxiety, boredom and depression (8–10). Therefore, it is of great practical significance to improve college students’ subjective well-being.

The key to improving college students’ subjective well-being lies in identifying its determinants. Subjective well-being is intricately tied to an individual’s living environment. School is the context in which children and adolescents pass almost one-third of their waking time, and it is the primary setting of social and emotional experiences as well as exposure to adverse events such as struggles with learning, peer rejection, bullying, and poor relationships with teachers (1, 11). As a consequence, schools play a privileged role in influencing their overall well-being (12). For college students, school remains the main context of life and learning, with learning being their principal endeavor. It follows that academic performance is inextricably linked to subjective well-being. Academic performance is an important reference index for college students indicated by awards, success in taking the postgraduate entrance examination to ensure postgraduate education, and study abroad, and it determines their future academic trajectories and employment (13). Research on middle school students has consistently shown that academic performance can significantly affect their subjective well-being (11, 14, 15).

However, the academic content, competitive pressure, and living environment differ markedly between college and middle school students, particularly since the outbreak of COVID-19 pandemic and its profound impact on educational systems. The extent to which academic performance is related to college students’ subjective well-being remains to be further studied.

A further gap in the literature concerns how academic performance is conceptualized. Most existing studies treat academic performance as an absolute metric, typically measured by GPA. However, in competitive educational environments, students do not evaluate their performance in isolation. Few studies have distinguished between absolute and relative academic performance when examining their associations with subjective well-being, leaving the independent role of class ranking largely unexplored.

Another notable limitation of existing research is the lack of systematic heterogeneity analysis. The association between academic performance and subjective well-being may not be uniform across student subgroups. Factors such as gender, grade level, and place of origin could moderate this relationship, yet prior studies have often treated student populations as homogeneous. Understanding these subgroup differences is crucial for designing targeted mental health interventions. Moreover, as recent research on Chinese university students has emphasized, studies should move beyond a single GPA-well-being association and consider a more ecological context that includes learning motivation, time management, classroom participation, self-regulation, institutional support, family background, and peer influence (16). This broader perspective helps identify which student subgroups are most vulnerable to performance-related distress and informs the design of more effective, context-sensitive interventions.

Thus, this study intends to take college students as the research object to explore the association between academic performance and their subjective well-being. The possible innovations of this study lie in the following. First, while existing research has largely focused on middle school students, we examine college students – a population whose academic environment, competitive pressures, and living context differ markedly from younger students. Second, beyond confirming the positive association between absolute academic performance (GPA) and well-being, we explicitly distinguish relative performance (GPA ranking) and demonstrate that ranking exerts an independent effect on well-being beyond raw GPA. Third, we systematically examine heterogeneity across gender, grade level, and place of origin, revealing that the well-being of female, lower-grade, and urban-origin students is more sensitive to academic performance. Fourth, our data were collected in October 2022, a period when COVID-19 restrictions were still actively shaping Chinese university life, providing a timely snapshot of student well-being under unique stressors.

## 2 Literature review

Subjective well-being encompasses a spectrum of pleasurable emotions that individuals experience, stemming from their perceptions of contentment and security. A multitude of factors impinge upon subjective well-being, with health widely acknowledged as the predominant influence (17–19). In addition, previous studies have also pointed out that exercise (20), digital disability (21) and information involvement (22) also significantly affect individuals’ subjective well-being. These studies mainly focus on adults or employees.

In recent years, students’ subjective well-being has attracted the attention of scholars. Students’ subjective well-being is classified into six dimensions: academic, psychological, self, physical, social and spiritual (4). Some scholars have designed an indicator system to measure students’ subjective well-being and analyzed the current situation and trends (1, 11, 23). Others have delved into the effects of various factors on students’ subjective well-being, including information processing style (24), school alienation (12), study major (25), mindfulness training (26), mathematics performance (27), physical activity (28), concerts (14) and family capital (29). However, the research mainly focuses on middle school students or primary school students, with relatively little attention paid to college students.

In terms of the relationship between academic performance and students’ subjective well-being, most studies have reported a positive association. For example, Morinaj and Hascher’s (12) longitudinal study of secondarly school students found that academic performance positively predicted subjective well-being over a one-year interval. Wu et al. (30) examined bidirectional relationships among Chinese middle school students and found that both present- and future-oriented subjective well-being were associated with later academic performance. However, these findings should be interpreted with caution due to several contextual factors. First, most of these studies focused on middle school or primary school students, whose academic environment, competitive pressure, and developmental stage differ substantially from college students. Second, the measures of subjective well-being varied across studies, ranging from single-item self-reports to multi-dimensional scales, which may affect the observed strength of associations. Third, the majority of existing research employed cross-sectional designs, which cannot establish temporal order or rule out reverse causality – it is equally plausible that students with higher subjective well-being perform better academically rather than the reverse.

Notably, a few studies have found no stable correlation between academic performance and students’ subjective well-being (31, 32). This inconsistency may reflect differences in sample characteristics: studies reporting null findings often involved college students or used different subjective well-being measures, whereas positive associations were more consistently found in younger populations. For instance, research on medical students has produced mixed results. Hagemeier et al. (33) found that medical students’ subjective well-being was negatively associated with the number of weekly examinations but positively associated with fall semester GPA. Monrad et al. (8) examined medical students and found that those with poorer well-being had lower Step 1 examination scores; however, after adjusting for Medical College Admission Test scores and cumulative GPA, the association was no longer significant. This suggests that academic aptitude and prior performance may confound the relationship between well-being and high-stakes exam outcomes, highlighting the importance of controlling for baseline academic ability – a consideration often overlooked in studies on this topic.

Taken together, the existing literature has three major limitations that motivate the present study. First, the evidence base is heavily skewed toward middle school and primary school populations, with limited research on college students despite their distinct academic and developmental contexts. Second, few studies have distinguished between absolute academic performance (GPA) and relative performance (GPA ranking), leaving the independent role of social comparison largely unexplored. Third, systematic heterogeneity analyses across gender, grade level, and place of origin are lacking, which is particularly important given that the association between academic performance and well-being may vary across student subgroups. The present study addresses these gaps by focusing on college students, distinguishing absolute from relative performance, and examining heterogeneity across key demographic dimensions.

## 3 Conceptual framework

Subjective well-being refers to individuals’ cognitive and affective evaluations of their lives, encompassing life satisfaction (34). In the context of higher education, understanding the determinants of college students’ subjective well-being is of paramount importance, as it is closely linked to their academic engagement, mental health, and long-term development (1, 2). Among the various factors that shape students’ subjective well-being, academic performance stands out as a particularly salient one, given the central role that academic achievement plays in the educational system, especially in China.

Several theoretical perspectives help explain why academic performance may exert a significant influence on college students’ subjective well-being. We focus on two complementary frameworks. First, Self-Determination Theory (SDT) (35) provides a well-established lens for understanding how absolute academic performance (GPA) may relate to well-being. SDT posits that the satisfaction of three basic psychological needs – autonomy, competence, and relatedness – is essential for intrinsic motivation and psychological wellness. In the academic context, high GPA can be interpreted as a marker of competence, one of the core needs in SDT. When students perceive that they are academically competent, their sense of mastery and effectiveness is enhanced, which in turn fosters greater well-being. Conversely, persistent academic difficulties may frustrate the need for competence, leading to diminished well-being (36). SDT has been extensively validated across educational settings and offers a parsimonious, empirically grounded explanation for the link between academic performance and well-being.

Second, Social Comparison Theory (37) explains why relative academic performance (GPA ranking) may be associated with well-being independently of absolute GPA. This theory suggests that individuals evaluate their own abilities and opinions by comparing themselves with others. In academic settings, students frequently engage in upward or downward comparisons with their peers regarding grades and rankings. Students with favorable class rankings may experience greater self-esteem and positive affect, while those with lower relative performance may suffer from feelings of inadequacy, anxiety, and diminished well-being. This comparative process is particularly pronounced in competitive educational environments, where academic standing is highly visible and socially consequential (38, 39). Notably, lower academic ranking may evoke not only social comparison effects but also shame, threats to perceived competence, and fear of negative evaluation from parents, teachers, and peers (40). Conversely, psychological resources such as self-compassion may help students process academic setbacks without an excessively sharp decline in well-being, a perspective that enriches the theoretical understanding of why some students are more resilient to low relative standing than others.

In addition to these two primary frameworks, we acknowledge Maslow’s Hierarchy of Needs Theory (41) as a broader motivational backdrop: academic performance can fulfill esteem and self-actualization needs, which may contribute to well-being (42, 43). However, SDT and Social Comparison Theory provide more direct and empirically testable mechanisms for the specific associations examined in this study.

We also acknowledge the Mindsponge Theory (44) as a complementary lens that emphasizes how individuals selectively absorb and process external information – such as academic feedback, parental expectations, and peer performance – within their sociocultural contexts. According to this theory, positive academic outcomes are more likely to be internalized as affirming one’s self-concept when they are perceived as beneficial or aligned with existing value systems, thereby enhancing well-being. Conversely, poor academic performance may be filtered as a threat to self-identity, leading to psychological distress (45, 46). However, we recognize that Mindsponge Theory is relatively recent and has not yet accumulated extensive independent empirical validation in the field of public health. Therefore, we use it here primarily as an illustrative framework to contextualize our findings within the broader information-processing perspective, rather than as a load-bearing theoretical pillar. Future research may further test its applicability in educational well-being studies.

In the Chinese context, the emphasis on academic performance is deeply embedded in the cultural and institutional fabric. Confucian heritage places a strong emphasis on educational achievement as a means of social mobility and family honor (29). Parents, teachers, and society at large often view academic success as a primary indicator of a student’s potential and worth. Consequently, students may internalize these external expectations, making their academic performance a critical determinant of their self-esteem and emotional well-being. Moreover, the highly competitive nature of the education system, exacerbated by the phenomenon of “involution” (neijuan) – a situation where excessive competition leads to diminishing returns – intensifies the psychological stakes associated with academic performance (47, 48).

Based on the above theoretical frameworks, we posit that both absolute and relative academic performance matter for college students’ subjective well-being. Absolute performance, as measured by GPA, reflects an individual’s objective level of achievement, which directly contributes to feelings of competence and self-worth. Relative performance, as captured by class rank, operates through social comparison mechanisms and may have an independent effect on well-being, particularly in competitive academic environments (consistent with Social Comparison Theory). Thus, we propose the following hypotheses:

*Hypothesis 1*: College students’ academic performance (GPA) is positively associated with their subjective well-being. The higher the GPA, the stronger the subjective well-being.

*Hypothesis 2*: College students’ relative academic performance (class ranking) is associated with their subjective well-being. Specifically, students with lower class rankings tend to report lower subjective well-being compared to those with higher rankings.

## 4 Methodology

### 4.1 Data

**Figure 1:**
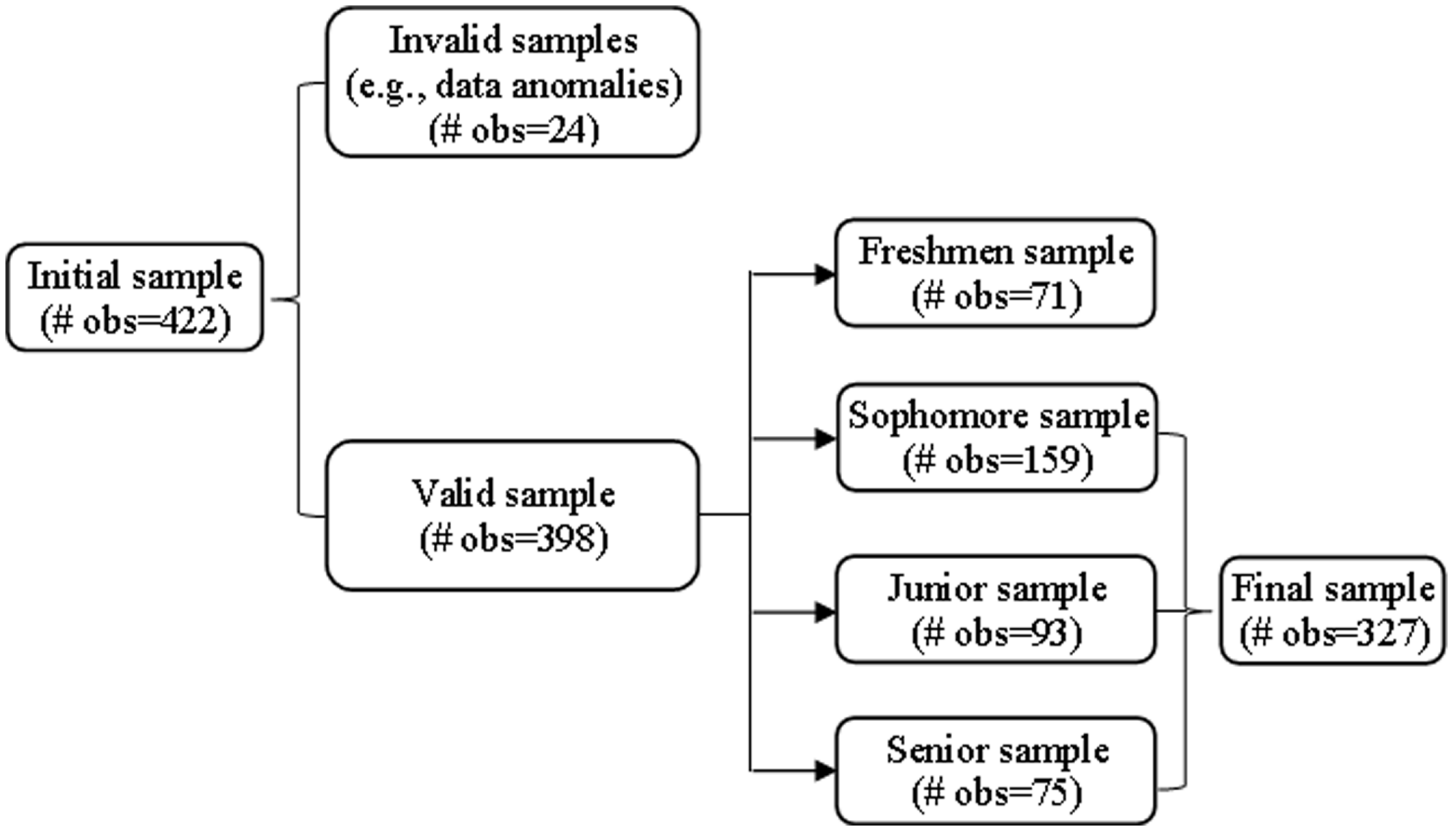
Sample selection.

The data used in this study come from the survey conducted by the research team in October 2022 with undergraduates at Beijing Technology and Business University. The questionnaire content included three parts: students’ basic characteristics, academic performance and individual cognition. We first designed a questionnaire around the research topic and conducted a preliminary survey at Beijing Technology and Business University. Based on the feedback from the survey, we revised and improved the questionnaire. After the formal questionnaire was designed, an electronic link was generated using the QuestionStar platform (a professional company that conducts online survey) and distributed through course group chats, WeChat Moments, and similar channels, encouraging current undergraduates of Beijing Technology and Business University to voluntarily and without compensation complete the survey. A total of 422 responses were collected through this online survey. For the purposes of this study, after excluding observations with missing data on key variables (i.e., the variables selected for the model) and invalid responses (e.g., implausible values such as age greater than 50 or negative GPA), 398 valid responses were retained, yielding a valid response rate of 94.3%. Since freshmen have no GPA, 71 freshman observations were excluded from the empirical analysis, resulting in a final analytical sample of 327 students. As shown in Figure 1, among these 327 students, sophomores, juniors, and seniors accounted for 48.62, 28.44, and 22.94%, respectively. In terms of academic fields, 58.41% were from the humanities and social sciences, and 41.59% were from the natural sciences. Additional sample characteristics are presented in Table 1.

**Table 1:** Variable definition and descriptive statistics.

Variable names	Variable definition	Min	Max	Mean
Dependent variable				
Subjective well-being	Self-rated subjective well-being (1–10 points)	1	10	6.875
Independent variable of interest				
Academic performance	GPA of last academic year	1.50	4.32	3.402
Control variables				
Grade	Sophomore = 1, junior = 2, senior = 3	1	3	1.743
Age	Age of college students (years)	18	26	20.177
Gender	Male = 1, female = 0	0	1	0.401
Major type	Major in social science = 1, major in science and engineering = 0	0	1	0.584
Post	Class-cadre = 1, not class-cadre = 0	0	1	0.462
Place of origin	Urban = 1, rural = 0	0	1	0.636
Family income	From extremely low to extremely high, assign values 1–5 in turn	1	5	2.899
Living expenses	1,000 yuan/month or below = 1, (1,000, 1,500 yuan/month) = 2, (1,500, 2000 yuan/month) = 3, (2000, 2,500 yuan/month) = 4, Above 2,500 yuan/month = 5	1	5	2.963
School club membership	Number of school clubs joined	0	5	1.101
Major preference	From extremely unlike to extremely like, assign values 1–5 in turn	1	5	3.459
Academic pressure	From extremely low to extremely high, assign values 1–5 in turn	1	5	3.431
Personality	From extremely introversion to extremely extroversion, assign values 1–5 in turn	1	5	3.080
Health	Self-rated physical health (1–10 points)	1	10	6.954

### 4.2 Variables

**Figure 2:**
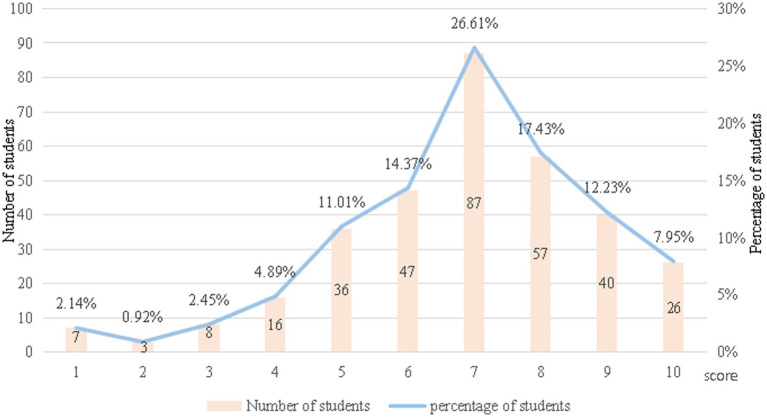
Students’ subjective well-being.

The dependent variable is college students’ subjective well-being. Although single-item measures cannot fully capture the multi-dimensional nature of subjective well-being, they have been widely used in large-scale educational surveys and researches. With references to previous researches (49–51), during the survey, students were asked to score their own subjective well-being, with values ranging from 1 to 10 as integers. The survey results showed that the average score of college students’ subjective well-being is 6.875, and the overall level of subjective well-being was not high. As shown in Figure 2, a total of 35.78% of the participants had a low level of subjective well-being, scoring less than or equal to 6 points. Only 37.61% of college students had a subjective well-being score of more than or equal to 8 points. In terms of grades, the sophomore students’ subjective well-being was the strongest, with an average score of 7.019. The junior and senior students’ subjective well-being was similar, with average scores of 6.667 and 6.827, respectively. In terms of gender, the average subjective well-being score of females was 6.934, higher than that of males (6.786), indicating that females had stronger subjective well-being than males, which is consistent with the findings of Raccanello et al. (1).

**Figure 3:**
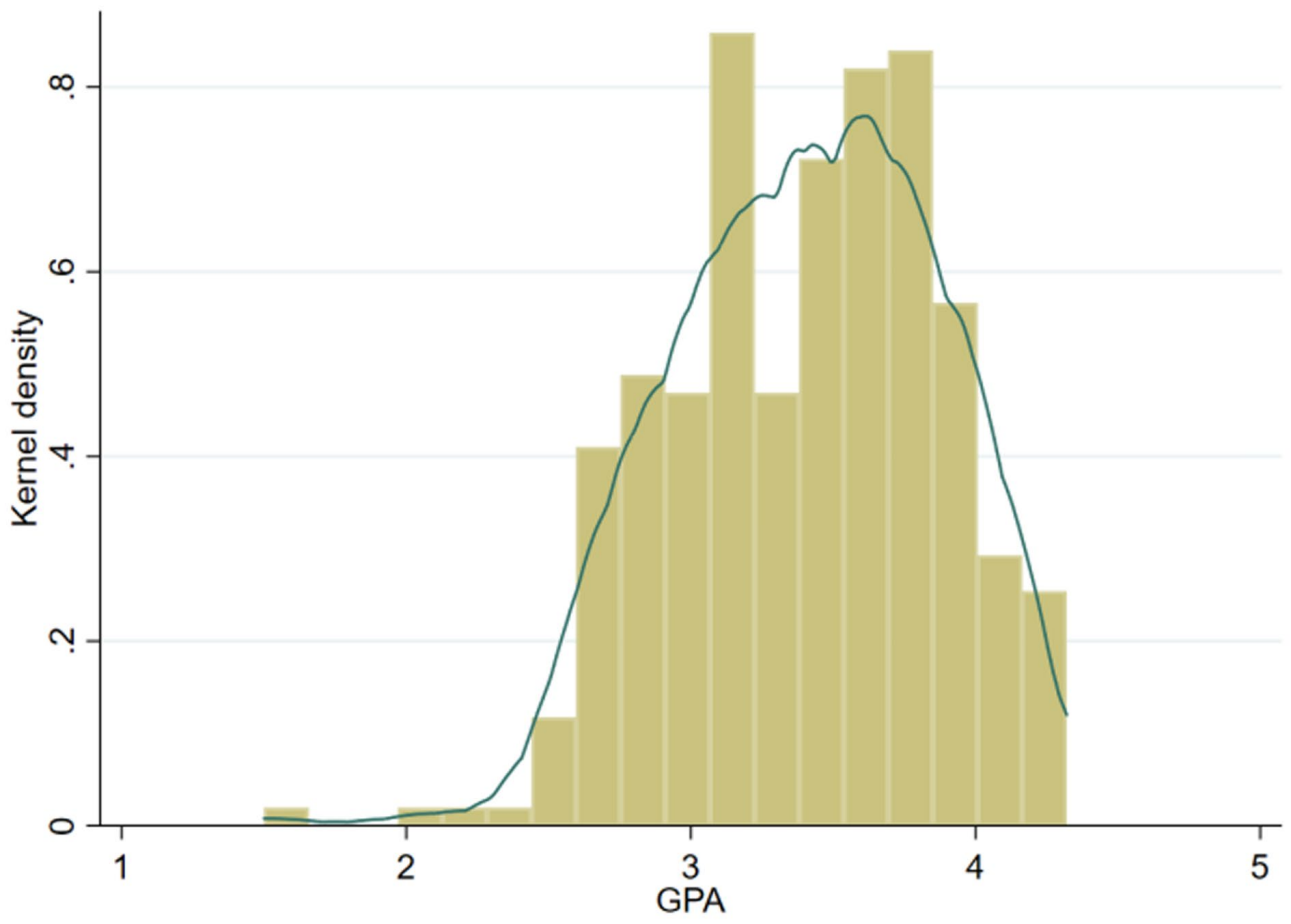
Kernel density estimation of students’ academic performance.

The independent variable of interest is college students’ academic performance. College students’ academic performance was measured by their GPA for the previous academic year, which is highly objective and representative. During the survey, students were asked to fill in the GPA of the previous academic year truthfully, and the value range was 0 ~ 5. While GPA was self-reported, students were assured of complete anonymity and were not required to provide identifying information, which encourages truthful responses. The survey results showed that the average GPA of participants was 3.402, which is above average (Figure 3) and closely related to the current “involution” social environment. The maximum GPA of college students is 4.32, the minimum is 1.50, and a few students failed in the exam.

With reference to existing studies (29, 52–54), variables such as participants’ year in school, age, gender, major, post, place of origin, family income, monthly living expenses, club participation in school, academic pressure, personality and health were also introduced to control the impact of other potential factors on their subjective well-being. See Table 1 for details of each variable.

### 4.3 Model

Since the subjective well-being of college students is assigned an integer of 1 ~ 10 from low to high, which belongs to an ordered variable, an ordered probit model (hereinafter referred to as Oprobit) is built. With reference to Wooldridge (55), the specific form of the model is as follows:


$$\mathrm{SW}B_{i}^{*}=\alpha +\beta_{1}\text{Performanc}e_{i}+\sum \text{φContro}l_{i}+\varepsilon_{i}$$ (1)

In Equation 1, $\mathrm{SW}B_{i}^{*}$ represents the latent variable of college students’ subjective well-being, and $\text{Performanc}e_{i}$ represents college students’ academic performance in the last academic year. $\text{Contro}l_{i}$ represents a series of control variables that may affect students’ subjective well-being. α , $\beta_{1}$ and φ represent the parameters to be estimated. $\varepsilon_{i}$ represents the residual disturbance term and is assumed to follow the standard normal distribution. The relationship between the observed value $\mathrm{SW}B_{i}$ and its latent variable $\mathrm{SW}B_{i}^{*}$ is as follows:


$$\mathrm{SW}B_{i}=\{\begin{array}{c} 1,\text{if}\;\mathrm{SW}B_{i}^{*}\leq C_{1}\\ 2,\text{if}\;C_{1}<\mathrm{SW}B_{i}^{*}\leq C_{2}\\ \ldots \ldots \ldots \ldots \\ 9,\text{if}\;C_{8}<\mathrm{SW}B_{i}^{*}\leq C_{9}\\ 10,\text{if}\;\mathrm{SW}B_{i}^{*}>C_{9} \end{array}$$ (2)

In Equation 2, $\mathrm{SW}B_{i}$ represents college students’ subjective well-being. $C_{1}$ ~ $C_{9}$ indicate cut points. When $\mathrm{SW}B_{i}^{*}<C_{1}$ , college students’ subjective well-being scores 1. When $C_{1}<\mathrm{SW}B_{i}^{*}<C_{2}$ , college students’ subjective well-being scores 2, and so on. The likelihood function of the sample can be obtained from Equation 2 above, and the maximum likelihood estimator, namely, the Oprobit model, can be obtained. Since the regression coefficient value of the Oprobit model does not have intuitive economic significance, we further calculate the marginal effect of each independent variable.

## 5 Results and discussion

### 5.1 The association between absolute academic performance and subjective well-being

In Table 2, regression (1) and regression (2) are the estimated results of the OLS model as a reference, and regression (3) and regression (4) are the estimated results of the Oprobit model, where regression (1) and regression (3) do not introduce control variables, and regression (2) and regression (4) introduce control variables at the same time. Whether or not control variables are introduced, OLS model estimation and Oprobit model estimation results show that the higher the college students’ GPA, the stronger their subjective well-being, which is consistent with the existing research conclusions for middle school students (11, 14, 15) and also provides support for hypothesis 1. From a theoretical perspective, this positive association aligns with Self-Determination Theory (35), which posits that the satisfaction of competence needs contributes to psychological well-being. Higher GPA can be interpreted as a marker of academic competence, and the observed effect, though modest in magnitude, supports this mechanism. In terms of effect size, the marginal effect of GPA on subjective well-being is 0.034 in the full model (Table 2). This means that a one-point increase in GPA is associated with a 0.034 increase in the probability of reporting a higher well-being category on the 1–10 scale. Given that well-being is influenced by many factors beyond academic performance, this effect size is comparable to those reported in meta-analyses of academic achievement and well-being (32). The other possible explanation for this association is that, first, the better the students’ academic performances are, the more chances they have to win scholarships and other related honors. Second, Chinese people attach great importance to education, and children’s academic performance is generally valued by parents, teachers and even the whole society (29). Therefore, academic performance tends to be recognized and praised by surrounding people, which may contribute to a student’s subjective well-being. Third, students often draw an equal sign between high academic performance and success; therefore, students, especially Chinese students, may view academic performance as a key factor associated with their subjective well-being (29). In addition, according to Maslow’s Hierarchy of Needs Theory (41), for college students, academic performance is the symbol of the realization of personal self-worth. If higher-level needs are met, higher subjective well-being tends to be reported. In short, a series of beneficial information may be generated around good academic performance, which, when internalized, may associated with better outcomes of subjective well-being (44, 50).

**Table 2:** Results of absolute academic performance’s impact on subjective well-being.

Variables	OLS model		Oprobit model (marginal effect)	
	Regression (1)	Regression (2)	Regression (3)	Regression (4)
Independent variable of interest				
Academic performance	0.698*** (0.227)	0.390** (0.165)	0.056*** (0.019)	0.034** (0.013)
Control variables				
Grade (sophomore is the reference group)				
Junior		−0.151 (0.191)		−0.018 (0.014)
Senior		0.198 (0.257)		0.011 (0.023)
Age		−0.067 (0.079)		−0.005 (0.007)
Gender		−0.423** (0.172)		−0.032** (0.014)
Major type		−0.132 (0.175)		−0.012 (0.014)
Post		−0.156 (0.158)		−0.017 (0.013)
Place of origin		−0.098 (0.165)		−0.003 (0.013)
Family income		0.162 (0.115)		0.011 (0.009)
Living expenses		−0.004 (0.088)		0.001 (0.007)
School club membership		0.127* (0.072)		0.011* (0.006)
Major preference		0.335*** (0.104)		0.028*** (0.008)
Academic pressure		−0.163 (0.121)		−0.014 (0.009)
Personality		0.161* (0.086)		0.014** (0.007)
Health		0.637*** (0.052)		0.051*** (0.008)
Constant	4.500*** (0.784)	1.151 (1.799)	–	–
*R* ^2^	0.028	0.537	–	–
Pseudo *R*^2^	–	–	0.008	0.192
Number of observations	327	327	327	327

The values in parentheses are robust standard errors. ***p < 0.01, **p < 0.05, *p < 0.1.

In terms of control variables, based on the estimated results of regression (4) in Table 2, the gender variable is significantly negative, indicating that the subjective well-being of males is lower than that of females, which is consistent with previous studies (56, 57). Because the emotional experience of females is richer and more profound than that of males, it is easier for them to feel a sense of belonging and dependence. In addition, it is easier for females to receive more support from friends and family than for males (58). Joining school clubs is significantly positive at the 10% statistical level, indicating that joining school clubs can improve students’ subjective well-being because school clubs organize activities that can enrich students’ spare time and provide more social opportunities. Clubs are often a source of social identification that can elicit subjective well-being (59, 60). The preference toward majors is significantly positive at the 1% statistical level, indicating that the more college students like their majors, the stronger their subjective well-being because doing what one likes makes one happy. The personality variable is significantly positive at the 5% statistical level, indicating that the more extroverted the students are, the stronger their subjective well-being, which is in line with the findings of Anglim et al. (61) and Lampropoulou (62). Because extroverted students have a wide range of communication activities and many ways to release emotions, they can digest negative energy and absorb positive energy in time. Moreover, extroverted students are better at expressing their feelings and needs and can also integrate into the collective faster and obtain more support, so they have better subjective well-being. The health variable is significantly positive at the 1% statistical level, indicating that the better the students’ physical health is, the stronger their subjective well-being, which is consistent with the literature (17–19). Good health is the basis for learning, entertainment and other activities, and it can create better living and learning conditions.

### 5.2 The association between relative academic performance and subjective well-being

The GPA measures the absolute academic performance of students. To further explore the impact of relative academic performance on students’ subjective well-being, we use GPA ranking to measure students’ relative academic performance. The questionnaire measures students’ relative academic performance by asking “What was your GPA ranking last academic year?”. Responses were “top 25%“, “top 25% ~ 50%“, “top 50% ~ 75%“and “bottom 25%“. Relative academic performance is used as the core explanatory variable to estimate the Oprobit model, and the estimated results are shown in Table 3. After introducing control variables, compared with students whose GPA ranked in the top 25%, the subjective well-being of students with GPA in the top 25% ~ 50 and 50% ~ 75% will be significantly lower, which may reflect the role of peer competition and academic pressure (38, 39, 63). In terms of the marginal effect, the subjective well-being of students with GPA in the top 50% ~ 75% will be reduced even more, which indicates that the lower the student’s GPA ranking is, the lower the student’s subjective well-being will be, and there will be a comparison effect. Hypothesis 2 is verified. This finding extends prior research that has largely focused on absolute academic performance, which is also consistent with Social Comparison Theory (37) and with studies showing that relative standing matters in competitive environments (38, 39). Students are concerned not only with absolute academic performance but also comparison with their classmates, that is, their GPA ranking. GPA in the bottom 25% shows no significant association with students’ subjective well-being. One possible explanation is that students whose GPA rank the lowest have a strong “lying flat” mentality – a colloquial term in contemporary China referring to a deliberate withdrawal from intense competition and a resignation to a low-effort, low-expectation lifestyle. However, it is important to emphasize that this interpretation is post-hoc and exploratory. Our survey did not directly measure academic disengagement, competitive attitudes, or coping strategies. Therefore, this explanation should be treated as a hypothesis for future research rather than a conclusive finding. Alternative explanations – such as measurement error, low statistical power due to the limited number of students in this category, or the possibility that bottom-ranking students attribute their performance to external factors (e.g., unfair grading) – cannot be ruled out. The effect sizes for ranking (marginal effects of −0.026 and −0.038 for the 25–50% and 50–75% groups, respectively) are small but meaningful, suggesting that even moderate downward comparisons can affect students’ emotional states.

**Table 3:** Results of relative academic performance’s impact on subjective well-being.

Variables	Oprobit model (Marginal effect)	
Independent variable of interest		
GPA ranking (students with GPA ranking top 25% is the reference group)		
Ranking top 25% ~ 50%	−0.069*** (0.023)	−0.026* (0.015)
Ranking top 50% ~ 75%	−0.074*** (0.023)	−0.038** (0.016)
Ranking bottom 25%	−0.093*** (0.025)	0.021 (0.041)
Control variables	Uncontrolled	Controlled
Pseudo *R*^2^	0.014	0.194
Number of observations	327	327

The values in parentheses are robust standard errors. ***p < 0.01, **p < 0.05, *p < 0.1.

### 5.3 Endogeneity test

Considering potential endogeneity issues, this study selects students’ college entrance placement test scores as an instrumental variable for their academic performance. In the questionnaire, this variable is measured by asking “How did your college entrance placement test score be?” with responses ranging from “very poor” to “very good,” coded as 1 to 5. Theoretically, college entrance placement test scores may satisfy both the relevance and exogeneity conditions for an instrumental variable. However, we acknowledge that the exogeneity assumption cannot be conclusively tested and should be interpreted with caution. On the one hand, entrance placement test scores effectively reflect students’ academic accumulation and basic abilities prior to college entry, and these ascriptive factors significantly influence their learning adaptability and course performance after enrollment. Therefore, there is a strong correlation between entrance placement test scores and college students’ academic performance, satisfying the relevance condition of the instrumental variable. On the other hand, entrance placement test scores represent a predetermined performance at the beginning of college, and it is a historical variable whose value does not change with students’ subsequent learning, social, or psychological states. Students’ current subjective well-being is primarily influenced by proximal factors such as academic stress, interpersonal relationships, and life satisfaction during college. As an early assessment, the entrance placement test score is unlikely to have a direct, independent causal effect on subjective well-being except through the indirect pathway of academic performance. Hence, this variable does not directly affect college students’ subjective well-being through channels other than academic performance, satisfying the exogeneity condition of the instrumental variable.

Since college students’ subjective well-being and relative academic performance are discrete variables, the Conditional Mixed Process (CMP) method is employed to test for endogeneity. The results are presented in Table 4, where CMP model (1) and CMP model (2) correspond to the tests using absolute academic performance and relative academic performance as the core explanatory variables, respectively. Taking the results of CMP model (1) for analysis, the instrumental variable is significant at the 1% statistical level in the first-stage regression, indicating a strong correlation between the instrumental variable and academic performance. In the second-stage regression results, after accounting for endogeneity, the effect of academic performance on college students’ subjective well-being remains significantly positive, providing additional support for the robustness of the observed association. Furthermore, this study finds that the endogeneity test parameter atanhrho_12 fails to reject the hypothesis that academic performance is an exogenous variable, suggesting that the above analysis of this study does not suffer from serious endogeneity issues, and meanwhile indicating that the model estimates are not significantly altered by the endogeneity test, which provides some reassurance regarding the stability of the findings. The results from CMP model (2) also corroborate the above conclusions. In addition, this analysis reduces but does not eliminate concerns about causal direction and omitted variables; the findings remain observational and should be interpreted accordingly. Future research using longitudinal designs and multi-informant measures would help address these limitations.

**Table 4:** Estimation results of endogeneity test.

Variables	CMP model (1)		CMP model (2)	
	First stage	Second stage	First stage	Second stage
Independent variable of interest				
Academic performance (GPA)		0.811** (0.366)		
GPA ranking (students with GPA ranking top 25% is the reference group)				
Ranking top 25% ~ 50%				−0.246* (0.144)
Ranking top 50% ~ 75%				−0.376** (0.161)
Ranking bottom 25%				0.163 (0.279)
College entrance placement test scores	0.159*** (0.025)		−0.329*** (0.047)	
Control variables	Controlled	Controlled	Controlled	Controlled
atanhrho_12	−0.228 (0.165)		0.246 (0.151)	
Number of observations	327	327	327	327

The values in parentheses are robust standard errors. ***p < 0.01, **p < 0.05, *p < 0.1.

### 5.4 Heterogeneity analysis

To further explore the differences in the impact of academic performance on different students, we divide the samples into six groups according to gender, grade and place of origin. First, students are divided into a female group and a male group according to gender. Second, sophomores are defined as the low-grade group, and junior and senior students are defined as the high-grade group. Third, according to the students’ place of origin, those who grew up in rural areas are defined as the rural group, and students who grew up in urban areas are defined as the urban group. The six sub-samples are estimated by the Oprobit model, and the estimation results are shown in Table 5.

**Table 5:** Results of heterogeneity analysis.

Variables	Grouped by gender (Marginal effect)	Grouped by grade (Marginal effect)			Grouped by place of origin (Marginal effect)	
	Female	Male	Low-grade	High-grade	Urban	Rural
Independent variable of interest						
Academic performance	0.033** (0.016)	0.026 (0.023)	0.045* (0.027)	0.021** (0.010)	0.046** (0.018)	0.028 (0.022)
Control variables	Controlled	Controlled	Controlled	Controlled	Controlled	Controlled
Pseudo *R*^2^	0.227	0.186	0.247	0.163	0.221	0.193
Wald chi^2^	107.21	103.67	85.98	98.34	118.19	96.88
Number of observations	196	131	159	168	208	119

The values in parentheses are robust standard errors. **p < 0.05, *p < 0.1.

From the perspective of gender, academic performance is significantly positively associated with females’ subjective well-being but shows no significant association with males’ subjective well-being. And the interaction test for gender is statistically significant as shown in Table 6, confirming that the positive association between academic performance and subjective well-being is significantly stronger for female students than for male students. Several tentative explanations may account for this gender difference. One possibility is that females behave better at school, are more gregarious (64), and they pay more attention to academic performance. Our survey data show that the average GPA of female students is 0.164 higher than that of male students. This is also a universal phenomenon in which female students show higher subjective well-being than males (23, 65, 66). Another possibility is that females may be more emotionally responsive to academic performance. In addition, the gender difference could be attributed to the individual’s mind-set, framed by exposure to life events, cultural values and beliefs influencing subjective judgments (44, 46). It should be noted that these explanations are speculative and should be treated as hypotheses for future research rather than conclusive findings.

**Table 6:** Results of interaction test.

Variables	Regression (1)	Regression (2)
Independent variable of interest		
Academic performance	0.369*** (0.016)	0.333*** (0.127)
Academic performance × Gender	−0.091** (0.039)	
Academic performance × Place of origin		−0.003 (0.038)
Control variables	Controlled	Controlled
Pseudo *R*^2^	0.192	0.192
Wald chi^2^	173.04	173.18
Number of observations	327	327

The values in parentheses are robust standard errors. ***p < 0.01, **p < 0.05.

From the perspective of grade, for both low-grade and high-grade students, the positive association between academic performance and subjective well-being appears to be stronger for low-grade students than for high-grade students. Junior and senior students, facing the choice of employment or further education, will no longer simply pursue a high GPA; as they move toward graduation, their attitude toward academic performance is relatively mature and stable. In addition, school adjustment, academic performance, and subjective well-being are stable over time (67). Thus, their academic performances appears less strongly associated with subjective well-being. For low-grade students, learning may be a more immediate priority goal in a short period of time and the most important way to compete with classmates. Academic performance may therefore be more strongly associated with their subjective well-being. In fact, grade-level differences exist in students’ subjective well-being, and subjective well-being generally decreases with increasing grade (1, 68, 69).

From the perspective of place of origin, for students growing up in urban areas, academic performance is significantly positively associated with their subjective well-being. The better their academic performance is, the stronger their subjective well-being. However, for students growing up in rural areas, their academic performances show no significant association with the subjective well-being of students from rural areas. As shown in Table 6, the interaction test for place of origin reveals that there is no significant difference between urban-origin and rural-origin students in the association between academic performance and subjective well-being. The apparent difference may be due to sampling variation rather than a true moderating effect of place of origin. According to the mindsponge theory, socio-cultural settings positively impact the individual’s evaluation of own health including mental health (45). Compared with urban living conditions, rural living conditions are relatively backwards, and a rural upbringing makes students more optimistic, mature and content (70, 71). The urban environment is more likely to cultivate students’ awareness of market competition, including studying. In addition, students from urban areas may experience greater competitive pressure than students from rural areas (72). Therefore, they may be more responsive to academic performance, and their subjective well-being may be more closely tied to academic outcomes. This result indirectly reflects the family capital differences affecting students’ subjective well-being (29). If future studies with larger samples were to find a difference, one speculative explanation might involve differential exposure to competitive environments, but this awaits empirical testing.

### 5.5 Robustness test

For the impact of absolute academic performance on students’ subjective well-being, we adopt two ways to test the robustness. First, the Tobit model is introduced for estimation, replacing the Oprobit model. Second, students’ satisfaction with the GPA of the previous academic year is used to measure academic performance. The core explanatory variable is changed, and the model is re-estimated. The estimation results are shown in the second and third columns of Table 6, respectively, which confirm that academic performance is significantly associated with students’ subjective well-being across alternative model specifications. For the impact of relative academic performance on students’ subjective well-being, the Tobit model is introduced, and we change the variable of subjective well-being from the “ten category” into the “five category” and re-estimate the Oprobit model. The estimation results are shown in the fourth and fifth columns in Table 7, which are completely consistent with the above empirical analysis results and prove the robustness of the results.

**Table 7:** Results of robustness test.

Variables	Tobit model	Oprobit model	Tobit model	Oprobit model
Independent variable of interest				
Academic performance	0.434** (0.179)	0.014** (0.007)		
GPA ranking (students with GPA ranking top 25% is the reference group)				
Ranking top 25% ~ 50%			−0.343* (0.196)	−0.051* (0.031)
Ranking top 50% ~ 75%			−0.524** (0.219)	−0.061* (0.033)
Ranking bottom 25%			0.252 (0.378)	0.028 (0.071)
Control variables	Controlled	Controlled	Controlled	Controlled
Pseudo *R*^2^	0.186	0.191	0.188	0.246
Number of observations	327	327	327	327

The values in parentheses are robust standard errors. **p < 0.05, *p < 0.1.

The COVID-19 pandemic context warrants special consideration. Data collection took place in October 2022, a time when many Chinese universities were still operating under strict health protocols, including online or hybrid learning, campus lockdowns, and limited social activities. These circumstances may have amplified academic stress and reduced opportunities for social support, potentially making more salient the observed association between academic performance and subjective well-being compared to pre-pandemic conditions. Conversely, the pandemic may have introduced additional sources of psychological distress that are independent of academic performance. Future research should examine whether the patterns reported here hold in post-pandemic educational environments.

## 6 Conclusion

### 6.1 Main findings

Subjective well-being is an important measure of college students’ quality of life, and it is also one of the multiple goals of college students’ training. Based on the survey data of undergraduates, we empirically examined the association between college students’ academic performances and their subjective well-being. The results show that the overall level of subjective well-being of college students is not high, at the lower middle level, and the level of subjective well-being of low-grade students is higher than that of high-grade students. Academic performance is significant positively associated with college students’ subjective well-being. The higher the college students’ GPA is, the stronger their subjective well-being. The GPA ranking of college students also significantly associated with their subjective well-being. The lower the GPA ranking is, the lower the subjective well-being. The results of sub-sample regressions show that academic performance is significantly and positively associated with females’ subjective well-being but shows no significant association with that of males. The positive association between academic performance and subjective well-being appears for both low-grade and high-grade students, and this association is stronger for low-grade students than for high-grade students. Likewise, academic performance is positively associated with the subjective well-being of students from urban areas, but shows no significant association with that of students from rural areas. In addition, gender, joining school clubs, major, personality and health also significantly affect college students’ subjective well-being. Specifically, the subjective well-being of female students is stronger than that of male students. The more school clubs students join, the more they like their majors, the more extroverted they are, and the healthier they are, the stronger their subjective well-being is.

### 6.2 Policy implications

These findings have important policy implications for promoting college students’ subjective well-being. First, the assessment methods of university education should be reformed and optimized. Schools should guide students to establish a correct concept of academic performance and avoid the influence of past academic performance on current emotions and later learning, teaching the students not to worry about gains and losses. In particular, given that GPA ranking exerts an independent effect on subjective well-being beyond raw GPA, universities should reconsider the practice of publicly ranking students. Reducing the salience of comparative performance information – for example, through private feedback mechanisms or grade non-disclosure policies – may be especially beneficial for students with lower relative standing.

Second, college students’ cognitive level of their majors should be improved. By incorporating specialized curriculum navigation courses or targeted lectures, students can be provided with a comprehensive overview of their major curriculum, insights into the job market, and an outlook on the future trajectory of their profession. This approach is designed to deepen their understanding of the field and stimulate a more profound interest in their academic pursuits. Schools should also improve the system of major diversion and transfer and open up channels for college students to choose their favorite majors.

Third, students should be encouraged to join school clubs based on their personal interests to enrich their college life. Schools should guide the outwards-oriented development of college students by organizing various group activities. Especially for students with more introverted personalities, more attention should be given to strengthen communication and encourage them to actively participate in class or club activities. This not only enhances their subjective well-being but is also beneficial for their future career development. Such efforts may be particularly important for female students and urban-origin students, whose subjective well-being is more sensitive to academic performance and who may therefore benefit more from alternative sources of social identification and self-worth.

Fourth, it is important to encourage college students to actively strengthen physical exercise. Health status is closely related to college students’ subjective well-being. According to our survey, the physical health of college students is not optimistic, with an average self-assessed health score of 6.95, and only 42.8% of students scoring 8 or above for health. Therefore, universities should pay attention to the construction of physical education courses and guide college students to develop the habit of exercising, and maintain good physical health.

### 6.3 Limitations and future directions

Several limitations of this study should be acknowledged. First, the sample was drawn from a single university located in Beijing, a metropolitan city in China. Consequently, the findings may not be directly generalizable to students from other institutional contexts, regions, or cultural settings. Future research should adopt multi-region, multi-institution sampling strategies to enhance external validity. Second, subjective well-being was measured using a single self-report item on a 1–10 scale. Although single-item well-being measures have been shown to possess adequate validity and are widely used in large-scale surveys, we acknowledge that they cannot capture the multi-dimensional nature of well-being as comprehensively as multi-item scales. Future studies may consider using validated multi-dimensional instruments. Third, GPA was self-reported by students rather than verified against official academic records. While we assured respondents of anonymity to encourage truthful reporting, the possibility of recall bias or social desirability bias cannot be entirely ruled out. Fourth, no *a priori* power analysis was conducted to determine the required sample size, particularly for subgroup analyses. The heterogeneity analysis involved sub-samples as small as 119 participants (e.g., rural-origin students). Non-significant findings in these subgroups should be interpreted with caution, as they may reflect insufficient statistical power rather than the absence of true effects. Finally, the data were collected in October 2022, a period when COVID-19-related restrictions and uncertainties were still actively shaping university life in China. While the pandemic context is not the focus of this study, it may have influenced students’ academic stress, social interactions, and subjective well-being in ways that are not fully captured in our analysis. Future research conducted in post-pandemic settings would help validate and extend our findings.

## Data Availability

The raw data supporting the conclusions of this article will be made available by the authors, without undue reservation.
